# HSV-2 glycoprotein gD targets the CC domain of tetherin and promotes tetherin degradation via lysosomal pathway

**DOI:** 10.1186/s12985-016-0610-7

**Published:** 2016-09-15

**Authors:** Yalan Liu, Mei Li, Di Zhang, Mudan Zhang, Qinxue Hu

**Affiliations:** 1 State Key Laboratory of Virology, Chinese Academy of Sciences, Wuhan Institute of Virology, Wuhan, 430071 China; 2 University of Chinese Academy of Sciences, Beijing, 100049 China; 3 Institute for Infection and Immunity, St George’s University of London, London, SW17 0RE UK

**Keywords:** HSV-2 glycoprotein D, Tetherin, Interaction, Lysosomal degradation

## Abstract

**Background:**

HSV-2 is the major cause of genital herpes. We previously demonstrated that the host viral restriction factor tetherin restricts HSV-2 release and is antagonized by several HSV-2 glycoproteins. However, the mechanisms underlying HSV-2 glycoproteins mediated counteraction of tetherin remain unclear. In this study, we investigated whether tetherin restricts the cell-to-cell spread of HSV-2 and the mechanisms underlying HSV-2 gD mediated antagonism of tetherin.

**Methods:**

Infectious center assays were used to test whether tetherin could affect cell-to-cell spread of HSV-2. Coimmunoprecipitation assays were performed to map the tetherin domains required for HSV-2 gD-mediated downregulation. Immunoflurence assays were performed to detect the accumulation of tetherin in lysosomes or proteasomes. All experiments were repeated for at least three times and the data were performed statistical analysis.

**Results:**

1) Tetherin restricts cell-to-cell spread of HSV-2; 2) HSV-2 gD specifically interacts with the CC domain of tetherin; 3) HSV-2 gD promotes tetherin to the lysosomal degradation pathway.

**Conclusions:**

Tetherin not only restricts HSV-2 release but also its cell-to-cell spread. In turn, HSV-2 gD targets the CC domain of tetherin and promotes its degradation in the lysosome. Findings in this study have increased our understanding of tetherin restriction and viral countermeasures.

## Background

Tetherin is an interferon (IFN)-inducible innate restriction factor involved in the host defense against the release of envelope viruses [1, 2]. Based on the deduced amino acid sequence, tetherin is characterized as a type II membrane protein with a Mr of approximately 24 kDa, while its mature form is a 30-36-kDa, heterogeneously glycosylated, dimeric, type II integral membrane protein, presumably due to heterogeneity of glycosylation during post-translational modification, comprising a small cytoplasmic tail (CT) domain, a trans-membrane (TM) domain, a glycophosphatidylinositol (GPI) membrane anchor at the C-terminus, and a long disulfide-rich coiled coil structure (CC) predicted for the extracellular domain [3, 4]. The mechanism by which different viruses counteract tetherin is distinct. To date, a number of viral proteins including Vpu and Env of HIV, Env and Nef of SIV, K5 of KSHV, Ebola GP, gM/VHS of HSV-1 and gB/gD/gH/gL of HSV-2 have been revealed to counteract the restrictive properties of tetherin [1, 5–12]. Except a recent report that Ebola GP may overcome tetherin restriction by blocking an interaction between VP40 and tetherin [13], the counteraction generally depends on the interaction between an viral antagonist and a specific domain of tetherin [14]. For instance, HIV-1 Vpu targets the TM domain of tetherin for the subsequent antagonism of tetherin function [1, 2, 15–19]; HIV-2 and tantalus SIV (SIVtan) Env interacts with the ectodomain of tetherin [20, 21], while other SIVs employ the Nef protein to target the cytoplasmic domain of tetherin [6, 8, 22]. The mechanisms underlying HIV-1 counteracts tetherin have been intensively investigated, but less attention has been paid to understanding those mediated by other viruses.

Herpes simplex virus 2 (HSV-2) is a DNA virus sexually transmitted and causes persistent infection that cannot be eliminated [23]. HSV-2 is the leading cause of genital ulcer disease (GUD) throughout the world and associated with human immunodeficiency virus 1 (HIV-1) acquisition [24–28]. HSV-2 transmission occurs via cell-free and direct cell-to-cell spread [29]. The HSV-2 glycoprotein D (gD), a major component of the virion envelope, was previously revealed to be essential for viral fusion and plays an important role in the cell-to-cell spread of the virus in permissive cells [30, 31].

Our previous study has revealed that tetherin functions as a restriction factor to inhibit HSV-2 release and that several glycoproteins including gD downregulate the expression of tetherin [11]. In this study, we investigated whether tetherin restricts the cell-to-cell spread of HSV-2 and the mechanisms underlying HSV-2 gD mediated antagonism of tetherin.

## Results

### Tetherin restricts cell-to-cell spread of HSV-2

**Fig. 1:**
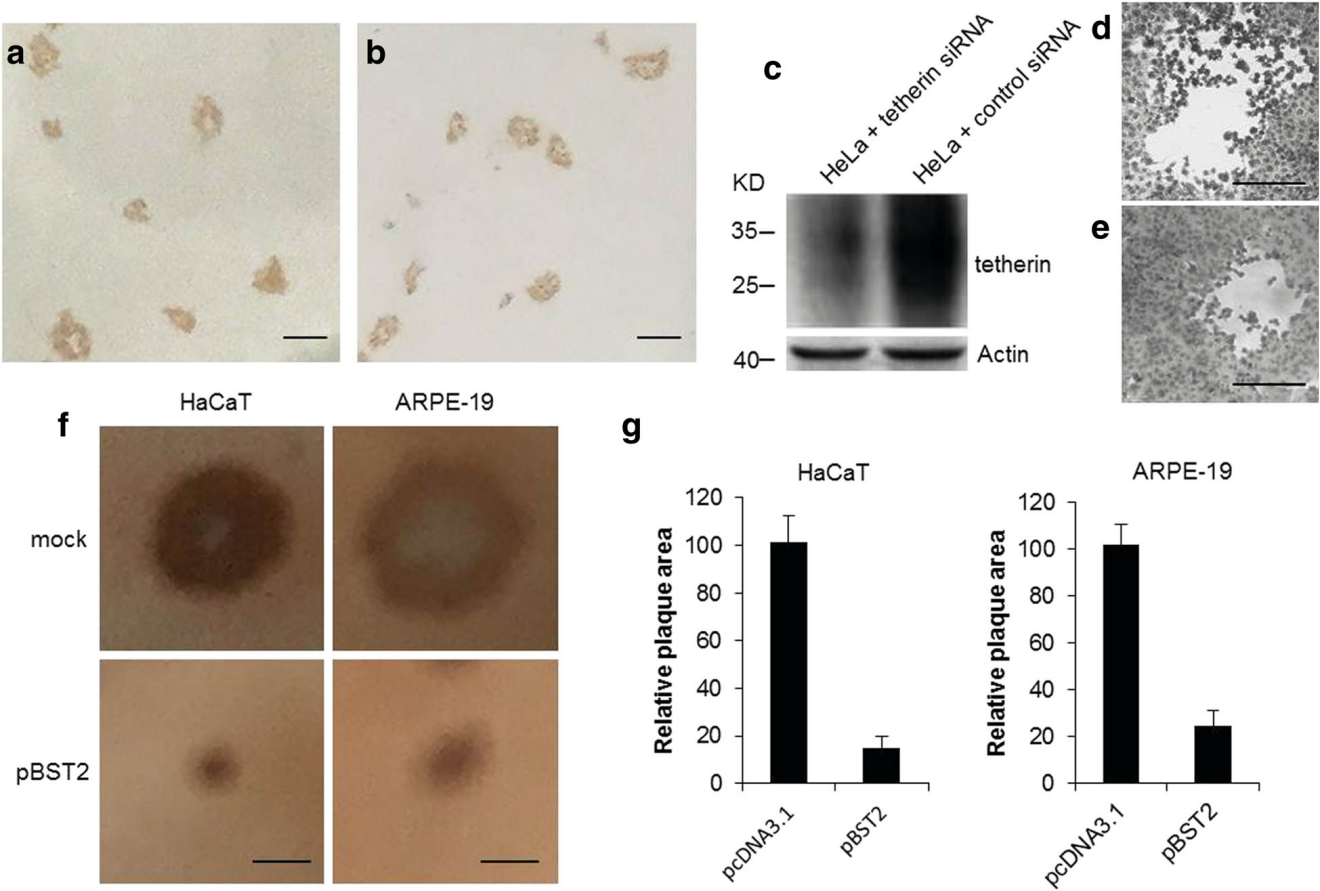
Tetherin restricts cell-to-cell spread of HSV-2. HeLa cells were infected with HSV-2 using 0.0001PFU/cell. Two hours later, the virus inoculum was removed and cells were incubated in the medium containing anti-HSV-2 antibody **a** or incubated in the normal medium without anti-HSV-2 antibody **b**. After 2 days, the cells in **a** and **b** were fixed and stained for HSV-2 antigens. The peroxidase-conjugated secondary antibody and substrate were used to reveal HSV-2-infected cells. **c** The expression of total tetherin in HeLa cells pretreated with tetherin siRNA or control siRNA was analyzed by western blot where actin was used as a loading control. Molecular weight standards in kilodalton are shown on the left. **d** and **e** The morphology of HSV-2 plaques on HeLa monolayers pretreated with tetherin siRNA **d** or control siRNA **e**. Representative fields observed in four experiments are shown. **f** The morphology of HSV-2 plaques on HaCaT and ARPE-19 monolayers transfected with pcDNA3.1 or pBST2 using the infectious center assay as described in the Materials and Methods. Representative morphology of HSV-2 plaques on HaCaT and ARPE-19 monolayers are shown. Scale bars in all panels represent 100 μm. **g** Representative fields containing more than 10 plaques were chosen and the plaque areas were calculated. The plaque area of the pcDNA3.1-transfected cells was arbitrarily set at a value of 100 for comparison with that of the pBST2-transfected cells. Data shown are mean ± SD

Spread of HSV-2 progeny virus can occur both by release of mature infectious virus particles into the extracellular medium and by viral cell-to-cell spread. Having demonstrated that the release of cell-free HSV-2 progeny virions was restricted by tetherin [11], we further asked whether tetherin could affect cell-to-cell spread of HSV-2 by using infectious center assay. Firstly, the plaque formation on HeLa monolayer with or without anti-HSV-2 antibody treatment was tested. The area of plaques was relatively uniform in the presence of anti-HSV-2 antibody, while some small spots existed in the samples without anti-HSV-2 antibody (Fig. 1a and b). The following experiments were all performed under the anti-HSV-2 antibody treatment. Subsequently, the HeLa monolayers pretreated with tetherin or control siRNA were examined. Western blot analysis indicated that siRNA knockdown of tetherin efficiently reduced the expression of tetherin (Fig. 1c). As shown in Fig. 1d and e, compared to control siRNA-pretreated HeLa cells, the plaque size was modestly increased upon tetherin siRNA pretreatment. Accordingly, two polarized epithelial cell types (HaCaT and ARPE-19) [32, 33] were used in the infectious center assay. As shown in Fig. 1f and g, the plaque size was significantly reduced on the pBST2 transfected HaCaT and ARPE-19 monolayers, confirming an interference with the cell-to-cell spread of HSV-2 in the presence of tetherin. Taken together, the reduced size of HSV-2 plaques by tetherin informs that tetherin at least partially inhibits the direct cell-to-cell spread of HSV-2 during plaque formation.

### HSV-2 gD specifically interacts with the CC domain of tetherin

**Fig. 2:**
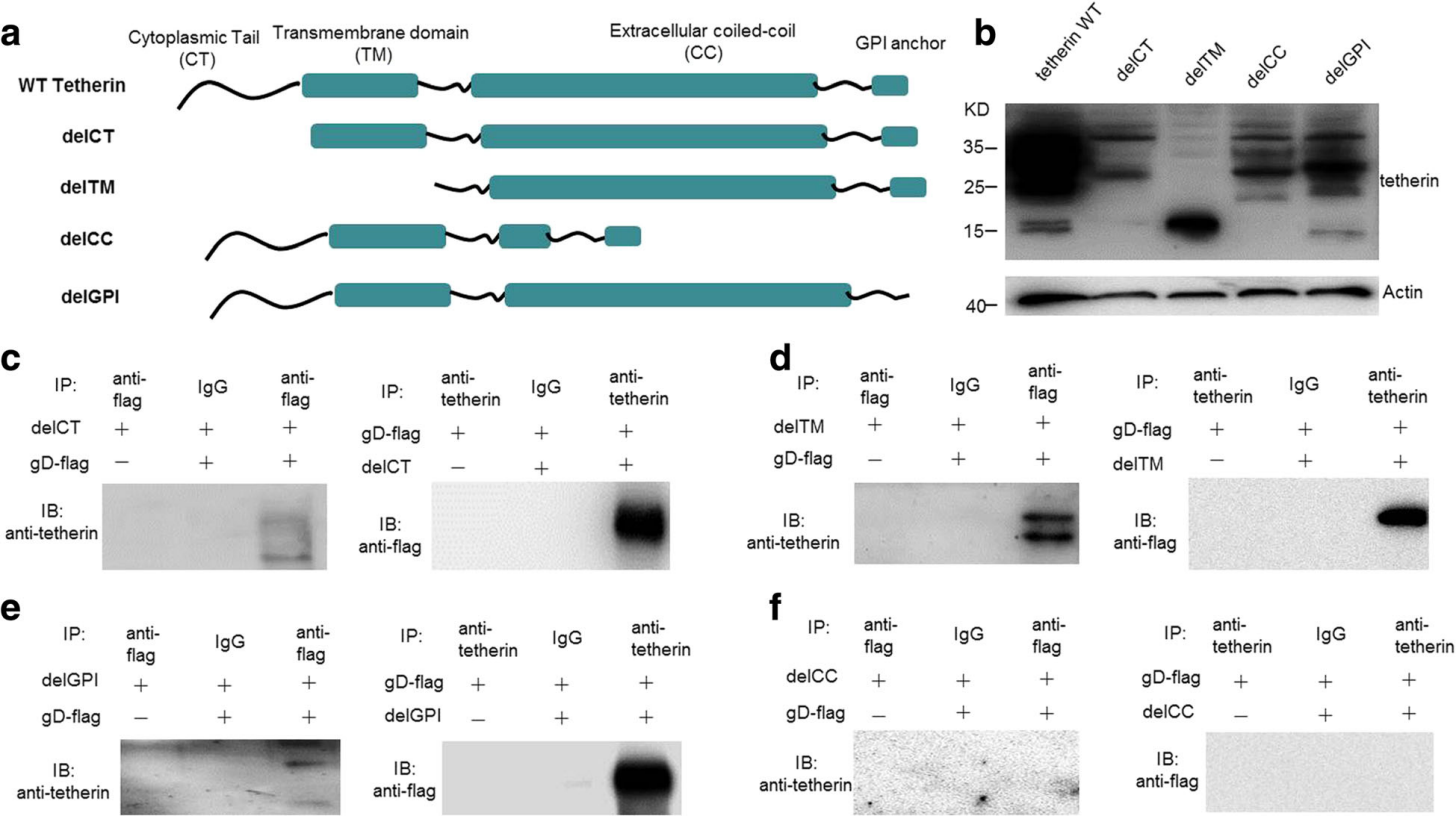
The CC domain of tetherin is specifically recognized by HSV-2 gD. **a** Schematic representation of full-length and deletion mutants of tetherin. The cDNAs of full-length human tetherin and its deletion mutants were cloned into pcDNA3.1(−), respectively. The TM, CC, and GPI domains of tetherin are represented in blue. **b** Expressions of WT and tetherin mutants were confirmed by western blot using the anti-tetherin polyclonal antibody. **c**-**f** Co-immunoprecipitation assays (co-IP) were performed by using the antibodies against tetherin and flag. The tetherin mutants delTM, delCT, delGPI and delCC were cotransfected with gD-flag into 293 T cells, respectively. The lysates were coimmunoprecipitated by the flag antibody and then western blot for tetherin (left panels) or coimmunoprecipitated by the tetherin antibody and then western blot for flag (right panels). **c** The interaction of gD-flag and the delCT. **d** The interaction of gD-flag and the delTM. **e** The interaction of gD-flag and the delGPI. **f** The interaction of gD-flag and the delCC

The interaction between HSV gD expressed in the progeny virus producing cells and the neighbouring host cells is essential for cell-to-cell spread. We previously demonstrated that several glycoproteins of HSV-2, including gD, can downregulate the expression of tetherin. To map the tetherin domains required for HSV-2 gD-mediated downregulation, we constructed a panel of deletion mutants of tetherin (Fig. 2a) and performed coimmunoprecipitation assays using cells transiently expressing wild type tetherin or its mutants. The expression of tetherin mutants was confirmed by western blot using an anti-tetherin antibody (Fig. 2b). Subsequently, the precleared cell lysates from the transfected cells were incubated with an anti-flag antibody or an isotype control antibody. The precipitates were analyzed by western blot using the antibody against tetherin. The tetherin mutants delTM, delCT and delGPI but not delCC were specifically coimmunoprecipitated by the flag antibody (Fig. 2c, d, e and f, left panels). Co-IP experiments were also performed by pulling down with the antibody against tetherin followed by western blot with the flag antibody. The antibody against tetherin was able to specifically precipitate the immune complexes that contained gD-flag and delTM, delCT or delGPI (Fig. 2c, d, e and f, right panels), indicating that only the CC domain of tetherin is required for the physical interaction with HSV-2 gD. Taken together, these data suggest that tetherin is down-regulated through a specific interaction between HSV-2 gD and the CC domain of tetherin.

### HSV-2 gD promotes tetherin to the lysosomal degradation pathway

**Fig. 3:**
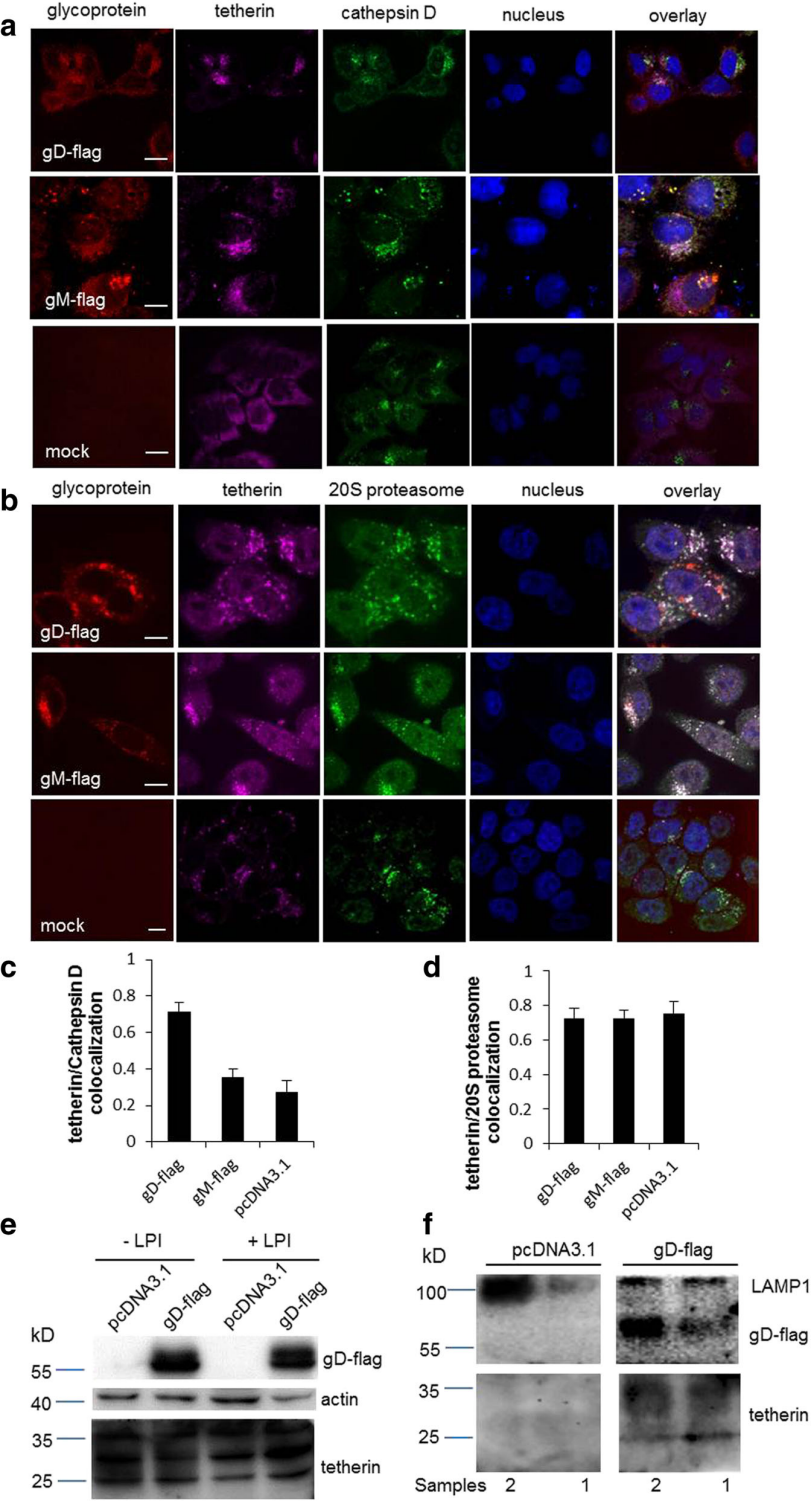
HSV-2 gD downregulates tetherin via lysosomal pathway. **a** HeLa cells transfected with pcDNA3.1 or plasmid expressing gD-flag/gM-flag were costained with anti-flag (red), anti-tetherin (purple) and anti-cathepsin D (green) antibodies. Nuclei were counterstained with Hoechst 33258 (blue). The colocalization of tetherin with lysosome markers (cathepsin D) was assessed by confocal microscopy. Representative confocal images from three independent experiments are shown. Scale bars in all panels represent 10 μm. **b** HeLa cells transfected with pcDNA3.1 or plasmid expressing gD-flag/gM-flag were costained with anti-flag (red), anti-tetherin (purple) and anti-20S proteasome (green) antibodies. Nuclei were counterstained with Hoechst 33258 (blue). The colocalization of tetherin with the proteasome markers (20S proteasome) was assessed by confocal microscopy. Representative confocal images from three independent experiments are shown. Scale bars in all panels represent 10 μm. **c** Pearson’s correlation coefficients were analyzed to determine the colocalization of tetherin and cathepsin D. **d** Pearson’s correlation coefficients were analyzed to determine the colocalization of tetherin and 20S proteasome. Data shown are mean ± SD by quantitative analyses of at least 20 distinct cells. **e** Western blot was used to analyze the expression of total tetherin in HeLa cells transfected with control plasmid and plasmid expressing gD-flag (treated or untreated with lysosome protease inhibitors (LPI)) where actin was used as a loading control. **f** HeLa cells transfected with pcDNA3.1 or plasmid expressing gD-flag were treated by lysis buffer. The prepared cell extract was ultracentrifuged by density gradient centrifugation and the lysosome band is located in the top 2 mL of the gradient. The corresponding bands were collected and the finally harvested lysosome pellets were detected by western blot. Samples 1 and 2 were two finally harvested lysosome pellets in two representative experiments

The known antagonists of tetherin can lead the degradation of tetherin in late endosomes, lysosomes or proteasomes [34]. To examine whether the gD mediated-downregulation of tetherin undergoes lysosomal or proteasomal degradation pathway, HeLa cells were transfected with pcDNA3.1 or gD expressing plasmid followed by cultivation in the presence of a mixture of lysosome protease inhibitors (LPI; containing leupeptin, pepstatin A, and E64d) or the proteasome protease inhibitor (PPI; containing MG132). The paralleled HeLa cells transfected with gM expressing plasmid were included as the control. At 24 hours post transfection, cells were processed for immunofluorescence staining for subcellular localization of tetherin, gD/gM and a lysosome marker cathepsin D or a proteasome marker 20S proteasome. As shown in Fig. 3a and c, colocalization of gD, tetherin and cathepsin D was seen in gD expressing cells treated with lysosomal protease inhibitors, whereas tetherin and cathepsin D did not apparently colocalize in the pcDNA3.1- or gM-transfected cells. Tetherin colocalized with the 20S proteasome in the presence or absence of HSV-2 gD (Fig. 3b and d), implying that tetherin likely physiologically undergoes proteasomal degradation in the absence of HSV-2 gD. Western blot assay demonstrated that the lysosome protease inhibitors (LPI) rescued tetherin from degradation in the gD-transfected cells (Fig. 3e). To confirm that the gD mediated-downregulation of tetherin undergoes lysosomal degradation, we used a lysosome enrichment kit (Thermo) to isolate and enrich the intact lysosomes from cells transfected with pcDNA3.1 or plasmids expressing gD. The prepared cell extract was ultracentrifuged by density gradient centrifugation. We collected the corresponding band and detected the finally harvested lysosome pellets by western blot. As shown in Fig. 3f, the lysosome marker LAMP1, tetherin and gD all existed in the separated samples from gD-transfected cells. In contrast, only LAMP1 existed in the samples from pcDNA3.1 transfected cells. These data together indicate that the gD mediated-downregulation of tetherin undergoes lysosomal degradation.

## Discussion

Tetherin has been shown to inhibit viral release and direct cell-to-cell spread of HIV-1 [35–37]. However, whether tetherin restricts cell-to-cell spread of other viruses is less clear. We previously showed that tetherin restricts viral release of HSV-2. Here we demonstrate that tetherin restricts the cell-to-cell spread of HSV-2, suggesting that such restriction may be physiologically relevant for viruses that are capable of cell to cell transmission. Interestingly, HSV-1 can use heparan sulfate (HS) as an entry receptor for infection of host cells [38]. However, during a productive infection, the HS moieties on parent cells can trap newly exiting viral progenies and inhibit their release and cell to cell spread. It has been revealed that heparanase (HPSE), a HS-degrading enzyme of the host, can translocate to the cell surface upon HSV-1 infection, leading to the removal of HS to facilitate viral release [39]. In agreement, potent HPSE inhibitor can successfully decrease cell-to-cell spread of HSV-1 and HSV-2 [40]. Although beyond the scope of this current study, it will be interesting to determine whether there is a similar enzyme or mechanism which can degrade tetherin to aid virus egress.

To counteract host restriction, viruses have evolved various evasion strategies. For instance, although tetherin efficiently inhibits the release of a number of enveloped virus particles by “tethering” them to the cell surface, different viral antagonists employ different mechanisms to counteract the restriction by tetherin. Current understanding how viruses counteract the restrictive property of tetherin is largely limited to the findings from retroviruses. In general, the counteraction is dependent of the interaction between viral antagonists and different domains of tetherin [14]. In the current study, we demonstrated that HSV-2 gD specifically interacts with the CC domain of tetherin.

There are several proposed mechanisms for the final fate of tetherin, including lysosomal degradation, proteasomal degradation, and/or sequestration/retargeting of tetherin to the trans-Golgi network [16, 21, 41–44]. Our previous study demonstrated that the levels of cell surface and total cellular tetherin were decreased in the presence of HSV-2 glycoproteins, implying that tetherin is likely counteracted by viral glycoproteins via a degradation pathway. In the current study, immunofluorescence images revealed that tetherin colocalized with the lysosome marker cathepsin D in the presence of HSV-2 gD, and western blot assay showed that gD and tetherin exist in the isolated lysosomes of gD-transfected cells, suggesting that gD promotes the degradation of tetherin in lysosomal pathway. Surprisingly, we found that the colocalization of tetherin and proteasome marker 20S proteasome was independent of the presence of gD, suggesting that tetherin itself, at least in HeLa cells, may physiologically undergo proteasomal degradation in the absence of HSV-2 gD. Although we cannot exclude other possibility, one possible explanation is that the proteasomal degradation may be cell-type dependent. Conjugation of proteins to ubiquitin is the hallmark of 26S proteasome mediated degradation, which is composed of a central 20S core containing catalytic subunits. The colocalization of tetherin and 20S proteasome implies the involvement of ubiquitinylation. Our data together demonstrated that tetherin indeed undergoes the lysosomal degradation in the presence of HSV-2 gD.

## Conclusions

We demonstrate that tetherin not only inhibits HSV-2 release but also restricts its cell-to-cell spread. In turn, HSV-2 gD targets the CC domain of tetherin and promotes its degradation in the lysosome. Findings in this study have increased our understanding of tetherin restriction and viral countermeasures.

## Methods

### Virus and cells

Human cervical epithelial cell line HeLa, human keratinocyte cell line HaCaT, African green monkey kidney cell line Vero and embryonic kidney cell line 293 T were grown in Dulbecco’s modified Eagle’s medium (DMEM; Gibco) containing 10 % FBS, 100 Units/ml penicillin and 100 Units/ml streptomycin at 37 °C in 5 % CO2. Human retinal pigment epithelial cell line ARPE-19 was grown in DMEM/F-12 medium (50:50) supplemented with 10 % FBS, 100 Units/ml penicillin and 100 Units/ml streptomycin at 37 °C in 5 % CO2. HSV-2 (G strain) was obtained from LGC standards and propagated in Vero cells. Virus stock was stored at −80 °C before used for infection.

### Plasmids

The open reading frames (ORF) of human tetherin amplified from pBST2 (Origene) was subcloned into pcDNA3.1(−) to construct the plasmid pWT (expressing full-length human tetherin). A panel of deletion mutants of tetherin were made and subcloned into pcDNA3.1(−), named delCT expressing 20–180 aa of human tetherin, delTM expressing 45–180 aa of human tetherin, delCC expressing human tetherin with the deleption of 100–154 aa and delGPI expressing 1–161 aa of human tetherin, respectively. The delTM without secretory leader peptide is not efficient for tetherin mutant to anchor on the cell surface membrane, we therefore fused the secretory leader peptide MDAMKRGLCCVLLLCGAVFVSPSQE from tissue plasminogen activator to tetherin codons 45–180 as described previously [45]. The glycoprotein gD and gM fused to flag tag were cloned into pcDNA3.1 (+) (named pgD-flag and pgM-flag) as described previously. The construction, cloning and propagation of plasmids were carried out using standard techniques. All constructs were verified by DNA sequencing (Sunny Biotechnology Co. Ltd, Shanghai, China).

### siRNA mediated knockdown of tetherin

Tetherin siRNAs (SI02777054) and control siRNA (1027281) were purchased from Qiagen. HeLa cells were plated onto 6-well culture dishes overnight. Hela monolayers were transfected with tetherin siRNA or control siRNA using HiPerFect Transfection Reagent (301704; Qiagen) according to the manufacturer's instructions.

### Infectious center assay

Infectious center assay was carried out as previously described [46]. In brief, HeLa, HaCaT and ARPE-19 cells plated in 6-well plates at 50 % confluence were exposed to HSV-2 at an MOI of 5 at 37 °C. After 90 min of incubation, cells were washed once with PBS and then treated with 0.1 M citrate buffer (pH 3.0) for 1 min to inactivate extracellular virus particles. The monolayer was then washed twice with PBS to remove the low-pH buffer, and cells were placed in growth medium supplemented with anti-HSV-2 antibody (PAB13979; Abnova) at a dilution of 1:1000 to neutralize extracellular HSV-2. After a total incubation of 5.5 h, the infected cells were detached with trypsin-EDTA, resuspended in growth medium, and ~100 cells were plated onto 50 % confluent monolayers of uninfected HaCaT and ARPE-19 cells transfected with pBST2 or pcDNA3.1, or of uninfected HeLa cells pretreated with tetherin or control siRNA. Cells were maintained in growth medium containing anti-HSV-2 polyclonal antibody. After 2 days, the cells were fixed and stained for HSV-2 antigens, followed by the addition of the peroxidase-conjugated secondary antibody and substrates. Plaques were photographed and the plaque areas were compared.

### Co-immunoprecipitation

Co-immunoprecipitation assay was carried out using Pierce Crosslink Immunoprecipitation Kit (26147; Thermo scientific) according to the manufacturer's instructions. For co-immunoprecipitations of gD-flag and tetherin mutants, mouse monoclonal antibody against FLAG (F1804; Sigma) and rabbit polyclonal antibody to tetherin (11721; NIH AIDS Research and Reference Reagent Program) were used for pull-down experiments. These two antibodies were also used for western blot analysis of the immunoprecipitates and the lysates.

### Western blot

Prepared cell lysates were resolved by 12 % SDS-PAGE and transferred to 0.45 μm polyvinylidene difluoride membranes (Millipore). Nonspecific binding was blocked using 5 % non-fat milk in PBS overnight at 4 °C. The membrane was incubated with primary antibody against tetherin (rabbit polyclonal antibody; 11721; NIH AIDS Research and Reference Reagent Program) at a dilution of 1:5000, FLAG (mouse monoclonal antibody; F1804; Sigma) at a dilution of 1:3000, and β-actin (mouse monoclonal antibody; sc-81178; Santa Cruz) at a dilution of 1:500, for 1 h at 37 °C. The membrane was washed five times with 0.1 % Tween 20/PBS, followed by incubation for 1 h with HRP conjugated goat anti–rabbit secondary antibody (1:10,000; BA1054, Boster) or HRP conjugated goat anti–mouse secondary antibody (1:10,000; BA1050, Boster). After five washes with 0.1 % Tween-20/PBS, the bands were visualized by exposure to FluorChem HD2 Imaging System (Alpha Innotech) after the addition of chemiluminescent substrate (SuperSignal® West Dura Extended Duration Substrate; 34075; Thermo Scientific Pierce).

### Immunofluorescence

To detect the accumulation of tetherin in lysosomes or proteasomes, transfected cells were cultured in complete medium in the presence of a mixture of lysosome protease inhibitors (containing leupeptin, pepstatin A and E64d; Sigma) for 16 h or a proteasome inhibitor (MG132; Sigma) for 12 h as previously described [16]. Transfected cells on 35 mm glass bottom culture dishes were washed twice with phosphate-buffered saline (PBS), followed by fixation with 4 % (w/v) cold paraformaldehyde for 30 min at room temperature. Cells were permeabilized with PBST (PBS-0.2 % (v/v) Triton X-100) for 10 min at room temperature and then blocked with PBS-2 % (w/v) BSA for 1 h at room temperature. Cells were incubated for 1 h at 37 °C with mouse monoclonal antibody against FLAG (F1804; Sigma) at a dilution of 1:200 and followed by incubation for 1 h at 37 °C with Cy3-conjugated goat anti-mouse secondary antibody (Beyotime, China) at a dilution of 1:200 in PBS-2 % (w/v) BSA. Thereafter, cells were incubated for 1 h at 37 °C with mouse monoclonal antibody against tetherin (H00000684-M15, Abnova) at a dilution of 1:200 and rabbit polyclonal antibody against cathepsin D (sc-10725; Santa Cruz) (or 20S proteasome α7/α8 (sc-67344; Santa Cruz)) at a dilution of 1:100 in PBS-2 % (w/v) BSA, followed by incubation for 1 h at 37 °C with Cy5-conjugated goat anti-mouse secondary antibody (Beyotime, China), FITC-conjugated goat anti-rabbit secondary antibody (Beyotime, China) at a dilution of 1:200 in PBS-2 % (w/v) BSA. Cells were washed three times after each incubation with PBS and then twice with distilled water. Nuclei were dyed by Hoechst 33258 (Invitrogen). Stained cells were analyzed using confocal microscopy (PerkinElmer UltraView VoX).

### Statistical analysis

All experiments were repeated for at least three times. Data are presented as mean ± SD unless otherwise specified. The difference of mean value was analyzed by a paired Student’s t-test. P < 0.05 was considered statistically significant.

## Abbreviations

CC: Coil-coiled
CT: Cytoplasmic tail
gD: Glycoprotein D
GPI: Glycophosphatidylinositol
GUD: Genital ulcer disease
HIV: Human immunodeficiency virus
HSV: Herpes simplex virus
MOI: Multiplicity of infection
TM: Transmembrane
WT: Wild-type
